# Choosing shoes: a preliminary study into the challenges facing clinicians in assessing footwear for rheumatoid arthritis patients

**DOI:** 10.1186/1757-1146-4-S1-O43

**Published:** 2011-05-20

**Authors:** Renee N Silvester, Anita E Williams, Nicola Dalbeth, Keith Rome

**Affiliations:** 1 Health & Rehabilitation Research Institute, AUT University, Auckland, 0627, New Zealand; 2 Directorate of Prosthetics, Orthotics and Podiatry, University of Salford, Salford, M6 6PU, UK; 3 Auckland District Health Board, Auckland, 1051, New Zealand; 4 University of Auckland, Auckland, 1010, New Zealand

## Background

Footwear has been accepted as a therapeutic intervention for the foot affected by rheumatoid arthritis (RA). Evidence relating to the objective assessment of footwear in patients with RA is limited. The aims of this study were to identify current footwear styles, footwear characteristics, and factors that influence footwear choice experienced by patients with RA.

## Methods

Eighty patients with RA were recruited from rheumatology clinics during the summer months from clinics based in Auckland, New Zealand. Clinical characteristics of RA, global function, and foot impairment and disability measures were recorded. Current footwear, footwear characteristics and the factors associated with choice of footwear were identified. Suitability of footwear was recorded using pre-determined criteria for assessing footwear type, based on a previous study of foot pain.

## Results

The patients had longstanding RA with moderate-to severe disability and impairment. The foot and ankle assessment demonstrated a low-arch profile with both forefoot and rearfoot structural deformities. Over 50% of shoes worn by patients were open-type footwear. More than 70% of patients' footwear was defined as being poor. Poor footwear characteristics such as heel rigidity and sole hardness were observed. Patients reported comfort (17%) and fit (14%) as important factors in choosing their own footwear. Only five percent (5%) of patients wore therapeutic footwear.

## Conclusions

The study found that moderate impairment and limited activity scores, consistent with significant foot disability. Forefoot structural deformities were high, suggesting that patients have problems in finding good footwear that accommodates structural changes in the forefoot and lesser extent in the rearfoot. Difficulties in finding appropriate footwear due to forefoot structural deformities and the consequence wearing of inappropriate footwear can be a major contributing factor to foot impairment. The study also demonstrated that although fit and comfort were perceived by patients to be important factors in choosing footwear, current footwear choices are frequently inappropriate. Choices regarding footwear may reflect the difficulties patients with RA experience when obtaining footwear that meets their needs. This work has highlighted the need for good footwear and the need to improve both patient and practitioner knowledge of footwear.

